# The 2016 Severe Floods and Incidence of Hemorrhagic Fever With Renal Syndrome in the Yangtze River Basin

**DOI:** 10.1001/jamanetworkopen.2024.29682

**Published:** 2024-08-22

**Authors:** Haoqiang Ji, Ke Li, Meng Shang, Zhenxu Wang, Qiyong Liu

**Affiliations:** 1 Department of Vector Control, School of Public Health, Cheeloo College of Medicine, Shandong University, Shandong Province, Jinan, People’s Republic of China; 2 National Key Laboratory of Intelligent Tracking and Forecasting for Infectious Diseases, National Institute for Communicable Disease Control and Prevention, Chinese Center for Disease Control and Prevention, Changping District, Beijing, People’s Republic of China; 3 World Health Organization Collaborating Centre for Vector Surveillance and Management, Changping District, Beijing, People’s Republic of China; 4 Department of Epidemiology, School of Public Health, Cheeloo College of Medicine, Shandong University, Shandong Province, Jinan, People’s Republic of China; 5 Shandong University Climate Change and Health Center, Shandong Province, Jinan, People’s Republic of China

## Abstract

**Importance:**

Hemorrhagic fever with renal syndrome (HFRS), a neglected zoonotic disease, has received only short-term attention in postflood prevention and control initiatives, possibly because of a lack of evidence regarding the long-term association of flooding with HFRS.

**Objectives:**

To quantify the association between severe floods and long-term incidence of HFRS in the Yangtze River basin and to examine the modifying role of geographical factors in this association.

**Design, Setting, and Participants:**

This cross-sectional study collected data on HFRS cases between July 1, 2013, and June 30, 2019, from 58 cities in 4 provinces (Anhui, Hubei, Hunan, and Jiangxi) in the Yangtze River basin of China, with a breakpoint of flooding in July 2016, generating monthly data. The 3 years after July 2016 were defined as the postflood period, while the 3 years before the breakpoint were defined as the control period. Statistical analysis was performed from October to December 2023.

**Exposures:**

City-level monthly flooding, elevation, ruggedness index, and closest distance from each city to the Yangtze River and its tributaries.

**Main Outcomes and Measures:**

The primary outcomes were the number of city-level monthly HFRS cases and the number of type 1 (spring or summer) and type 2 (autumn or winter) HFRS cases.

**Results:**

A total of 11 745 patients with HFRS were reported during the study period: 5216 patients (mean [SD] age, 47.1 [16.2] years; 3737 men [71.6%]) in the control period and 6529 patients (mean [SD] age, 49.8 [15.8] years; 4672 men [71.6%]) in the postflood period. The pooled effects of interrupted time series analysis indicated a long-term association between flooding and HFRS incidence (odds ratio, 1.38; 95% CI, 1.13-1.68), with type 1 cases being at highest risk (odds ratio, 1.71; 95% CI, 1.40-2.09). The metaregression results indicated that elevation and ruggedness index were negatively associated with the risk of HFRS, while the distance to rivers interacted with these associations.

**Conclusions and Relevance:**

This cross-sectional study of the long-term association between flooding and HFRS incidence, as well as the modification effects of geographical factors, suggests that severe floods were associated with an increased risk of HFRS within 3 years. This study provides evidence for the development of HFRS prevention and control strategies after floods.

## Key Points

**Question:**

Were severe floods in China in 2016 associated with the incidence of hemorrhagic fever with renal syndrome (HFRS), and did geographical factors modify this association?

**Findings:**

In this cross-sectional study of 58 cities in China and 11 745 patients with HFRS, severe floods in 2016 were associated with an increased risk of HFRS within 3 years. Geographical factors played a role in modifying the association between severe floods and HFRS incidence.

**Meaning:**

This study suggests that China should not only focus on short-term HFRS prevention and control measures after floods but also prioritize long-term flood prevention and control efforts.

## Teaser

This cross-sectional study examines the association between severe floods and long-term incidence of hemorrhagic fever with renal syndrome in the Yangtze River basin and explores the modifying role of geographical factors in this association.

## Introduction

Although hemorrhagic fever with renal syndrome (HFRS) is a widely distributed zoonotic disease globally, it has been neglected by many governments and the public in recent years.^1^ Patients are infected mainly through contact of damaged skin or mucous membranes with aerosolized urine, droppings, or saliva of rodents carrying the hantavirus or by exposure to dust from their nests or bedding.^2^ It is called by different names in different continents due to the different organs affected by various serotypes of the virus: hantavirus pulmonary syndrome in the Americas and HFRS in Europe and Asia.^3,4^ In the past few decades, China has accounted for more than 90% of all HFRS cases worldwide (576 361 cases from 1995 to 2020), with the highest fatality rate reaching 15%.^5,6^ In the past decade or so, both the incidence of HFRS and the mortality rates from HFRS have decreased due to improved health conditions, with the national mean incidence rate at approximately 1 per 100 000 population and that a fatality rate at around 1%.^7^ However, HFRS still poses a potential threat of outbreaks and resurgence globally due to global climate changes. Prioritizing HFRS prevention and control efforts remains a significant concern for public health worldwide.^8^

In the context of global climate change, the frequency and intensity of floods are gradually increasing, which cause more serious damage to China.^9,10^ Such floods not only lead to direct loss of lives and infrastructure damage but also increase the likelihood of emerging and reemerging infectious diseases, including HFRS.^11,12,13,14,15,16^ However, China has focused on only short-term HFRS prevention measures after floods.^17,18,19^ Insufficient evidence regarding the long-term effects of rodent populations on the risk of HFRS makes it difficult for frontline practitioners to develop rodent eradication strategies after floods. In terms of cascade effects, higher rodent densities accelerate the transmission of hantavirus among rodents, and the level of infections increases with the age and number of offspring, continuously increasing the long-term risk of human infection.^20^ Therefore, it is crucial to examine the association of floods with HFRS transmission so that effective prevention strategies can be developed.

As a rodent-borne disease with rodents as a natural reservoir, hydrologic conditions may have a greater association than temperature with transmission of HFRS.^21,22,23^ Previous studies have mostly demonstrated a positive association between hydrologic conditions and HFRS incidence,^13,14,15^ using time series data analyzing the lagged effects of extreme precipitation on HFRS incidence within a 6-month period.^4,24,25,26^ Studies have found that exposure to floods could lead to an increased short- and medium-term HFRS risk^27,28^ and changes in rodent population density. However, previous studies neglected the ecological characteristics of rodents, as floods can disrupt their habitats, leading to the aggregation of different rodent species in safer areas, such as residential zones and highlands. This disruption of rodent habitats could result in short-term changes in HFRS. When different rodent species aggregate and reproduce multiple generations in these newly established safe areas, it may pose a long-term threat to the health of the population in that region (eFigure 1 in Supplement 1). Therefore, this study used the severe flood events in the Yangtze River in 2016 to validate the long-term association between flooding and the incidence of HFRS.

## Methods

### Severe Flood Event

This study selected the severe flood event in the Yangtze River basin of China, which occurred from June 30 to July 6, 2016, caused by El Niño. The event was the most severe meteorological disaster in China since 1998. The 2016 floods affected 55.5 million people in the study area (with a population of 20.8 million in Hubei Province, 15.3 million in Hunan, 12.8 million in Anhui, and 6.6 million in Jiangxi), resulting in 206 deaths and direct economic losses of ¥171.02 billion. Based on the definition in the 2017 Yearbook of Meteorological Disaster in China and local meteorological disaster yearbooks, it was determined whether each city encountered floods as a binary variable (eFigure 2 in Supplement 1; 41 of 58 cities were flood-affected areas).^29^ To explore the risk of HFRS incidence within 3 years after flooding, this study set the time span from July 1, 2013, to June 30, 2019, with a breakpoint in July 2016, generating monthly data. The 3 years after July 2016 were defined as the postflood period (assigned a value of 1), while the 3 years before the breakpoint were defined as the control period (assigned a value of 0). All personally identifiable information of the cases, such as names and ID, was deidentified. Thus, this study was deemed exempt from ethical approval, and the informed consent requirement was waived by the Shandong University institutional review board. This study followed the Strengthening the Reporting of Observational Studies in Epidemiology (STROBE) reporting guideline.

### HFRS Cases

The data on HFRS cases were sourced from the Infectious Disease Reporting Information Management System of the Chinese Center for Disease Control and Prevention. The diagnostic criteria and clinical features for HFRS cases were based on the diagnostic criteria for epidemic hemorrhagic fever issued by the National Health Commission of China^30^ (eMethods in Supplement 1). The individual case information mainly includes data such as age, gender, occupation, current address, onset date, diagnosis date, and death date. The inclusion and exclusion criteria for HFRS cases are shown in eFigure 3 in Supplement 1.

In this study, the HFRS case data were grouped and summarized into monthly databases by study area. According to the seasonal patterns of HFRS incidence, type 1 cases are mainly caused by *Rattus norvegicus* in spring and summer (March to August), while type 2 cases are mainly caused by *Apodemus agrarius* in autumn and winter (September to February).^26,31^ The administrative codes (eTable 1 in Supplement 1) of the study area in 2019 were used to match the case codes, and any mismatched data were corrected on a case-by-case basis before matching.

### Geographical Factors

The map of China was derived from the National Platform for Common Geospatial Information Services, with an approved number of GS (2019) 1822. The elevation raster data, with a resolution of 500 m, and the third-level river shapefile were obtained from the Resource and Environment Science and Data Center of the Chinese Academy of Sciences (eFigure 4 in Supplement 1^32^). The mean elevation (in kilometers) of each city was extracted using raster data, and the ruggedness index was the SD of city elevations. The “spdep” package in R, version 4.2.2 (R Project for Statistical Computing) was used to calculate the distance (in kilometers) from the center of each city to the Yangtze River and its tributaries.

### Statistical Analysis

Statistical analysis was performed from October to December 2023. The characteristics of the HFRS cases were described as numbers and percentages for categorical variables, mean (SD) values for normally distributed data, and median (IQR) values for nonnormally distributed data. To compare the differences, *t* tests were conducted for continuous variables, χ^2^ tests for categorical variables, and the Kruskal-Wallis test for other variables. The study described the temporal trends and spatial distribution of HFRS incidence. In addition, the spatial autocorrelation of the case distribution was analyzed using the Moran test.

Interrupted time series analysis is a statistical method used to assess the association of an intervention with time series data. It helps determine whether the intervention measurements were associated with significant changes in disease incidence and excludes other possible factors that may have affected the results.^33^ In this study, interrupted time series analysis models with a quasi-Poisson distribution as the link function were used to validate the association between floods and HFRS incidence. Considering that HFRS has 2 peaks annually, Fourier terms (order = 2) were introduced into the models to adjust for the seasonality of HFRS incidence, and monthly factor variables along with continuous month variables for each period were also included to control for monthly effects and long-term linear trends. After conducting the Durbin-Watson test (step 1), if the Durbin-Watson value significantly deviated from 2, it indicated the presence of autocorrelation in the residuals, which may affect the accurate estimation of the model. Therefore, if this problem occurred, a lagged residual term would be added to the model (step 2). In addition, subgroup analyses for each region were conducted to explore the risk of HFRS after flooding. The odds ratio (OR) and 95% CI represent the risk of HFRS within 3 years after flooding. Finally, the study used generalized additive models for sensitivity analysis.

Meta-analysis was used to explore the pooled effect of the risk of HFRS incidence after flooding, thereby controlling for spatial heterogeneity across different regions. First, random-effect meta-analyses were conducted for flood and nonflood cities, and heterogeneity was evaluated using the *I*^2^ statistics and the *Q* test. Second, if high heterogeneity was observed (*I*^2^ > 20% and *P* < .10 for *Q* test), sensitivity analysis was conducted by excluding the region with the highest heterogeneity. Then, fixed-effect meta-analyses were performed to estimate the pooled effect of groups with low heterogeneity. Subgroup analyses based on provinces were conducted for areas with high heterogeneity, following the same steps as before. Third, meta-analyses were conducted to examine the pooled effects of the risk of type 1 and type 2 cases separately. These strategies were repeated for these 2 categories. In addition, metaregression analyses were conducted to examine the associations among elevation, ruggedness index, city’s distance to rivers, and the risk of HFRS after flooding. All analyses were implemented in R, version 4.2.2 (R Project for Statistical Computing). All *P* values were from 2-sided tests and results were deemed statistically significant at *P* < .05.

## Results

### Characteristics of HFRS Cases

**Figure 1.**
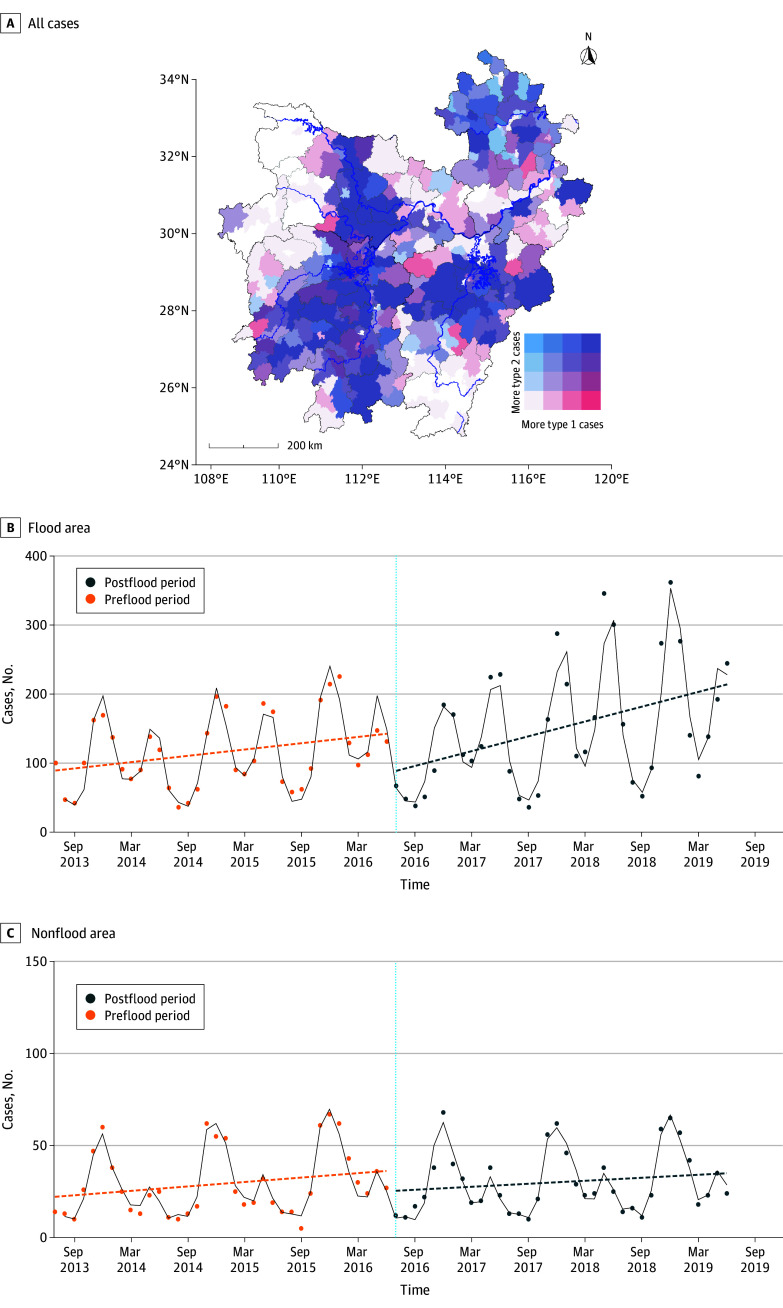
Spatial Distribution and Time Trend of Hemorrhagic Fever With Renal Syndrome Cases From July 2013 to June 2019 The scatterplots represent the actual values, the dashed line represents the fitted line of the actual values, and the black line represents the model’s curve.

From July 2013 to June 2019, a total of 11 745 HFRS cases were reported in the study area. In the control period, there were 5216 cases (mean [SD] age, 47.1 [16.2] years; 3737 men [71.6%]), accounting for 44.4% of cases. In the postflood period, there were 6529 reported cases (mean [SD] age, 49.8 [15.8] years; 4672 men [71.6%]), accounting for 55.6% of total notified cases. During the study, 70.6% of the patients (8291 of 11 745) were farmers. After flooding, the main increase was observed in type 1 cases (3129 of 5325 [58.8%]). The percentage of cases in the flood areas was higher after flooding (5442 of 6529 [83.4%]) compared with the nonflood areas (*P* < .001). Other characteristics of the cases can be found in eTable 2 in Supplement 1. During the study period, HFRS cases exhibited a bimodal distribution, with higher peaks in type 2 cases compared with type 1, except within the 2 years after floods. eFigure 5 and eFigure 6 in Supplement 1 illustrate the temporal trends in HFRS cases, categorized by flooding occurrence and province, respectively. In addition, HFRS cases were distributed mainly near rivers, and all types of HFRS showed certain spatial clustering (Moran *I*, 0.299-0.307; Figure 1A).

### Results of the Interrupted Time Series Analysis

According to Table 1, after adding a lagged residual term (step 2), the Durbin-Watson values gradually approached 2, indicating a better performance at estimating outcomes compared with the results of step 1. The risk of HFRS incidence increased within 3 years after flooding (OR, 1.27; 95% CI, 1.17-1.39; *P* < .001). This risk was more pronounced in the flood areas (OR, 1.33; 95% CI, 1.20-1.47), while no significant association was observed in nonflood areas (OR, 1.06; 95% CI, 0.98-1.15; *P* = .17). eFigure 7 in Supplement 1 shows that the diagnostic results of the interrupted time series analysis models demonstrate a good fit. Figure 1 shows that, after flooding occurred, type 2 cases initially decreased in the first year in the flood areas, while type 1 cases surpassed type 2 cases for the first time. This phenomenon lasted for about 2 years, and in the third year after flooding, type 2 cases continued to increase while type 1 cases decreased. In addition, the number of HFRS cases in flood areas significantly increased after flooding, with a steeper slope compared with the control period. However, this phenomenon was not observed in nonflood areas (eFigure 8 in Supplement 1). With the use of the generalized additive model for sensitivity analysis, the results showed that as the lag period of flooding increased, the risk of HFRS incidence initially decreased, then fluctuated and increased, followed by a slow decrease (generalized cross-validation = 5.298; *P* < .001). However, the results of the model for nonflood areas were not statistically significant (generalized cross-validation = 1.240; *P* = .12). This is consistent with the findings of the study. (eFigure 9 in Supplement 1).

**Table 1.** Risk of HFRS Within 3 Years After Flooding

Study area	Results of step 1^a^		Results of step 2^b^		
	DW value	OR (95% CI)	DW value	OR (95% CI)	*P* value
**All types of HFRS**					
All areas	3.71	1.26 (0.96-1.66)	1.85	1.27 (1.17-1.39)	<.001
Flood area	0.79	1.31 (1.16-1.45)	1.72	1.33 (1.20-1.47)	<.001
Nonflood area	2.21	1.05 (0.97-1.14)	1.87	1.06 (0.98-1.15)	.17
**Type 1 HFRS**					
All areas	1.12	1.24 (1.13-1.35)	2.08	1.31 (1.21-1.42)	<.001
Flood area	1.11	1.27 (1.15-1.40)	2.14	1.36 (1.24-1.49)	<.001
Nonflood area	1.48	1.04 (0.96-1.12)	1.80	1.05 (0.98-1.14)	.19
**Type 2 HFRS**					
All areas	1.42	1.14 (1.04-1.24)	1.78	1.14 (1.04-1.25)	.006
Flood area	1.20	1.16 (1.04-1.29)	1.62	1.17 (1.05-1.30)	.004
Nonflood area	2.50	1.05 (0.95-1.15)	1.87	1.05 (0.96-1.14)	.34

Abbreviations: DW, Durbin-Watson; HFRS, hemorrhagic fever with renal syndrome; OR, odds ratio.

^a^ Conducting the DW test.

^b^ Adding lagged residual term to the model.

### Pooled Effects of the Risk of HFRS After Flooding

**Figure 2.**
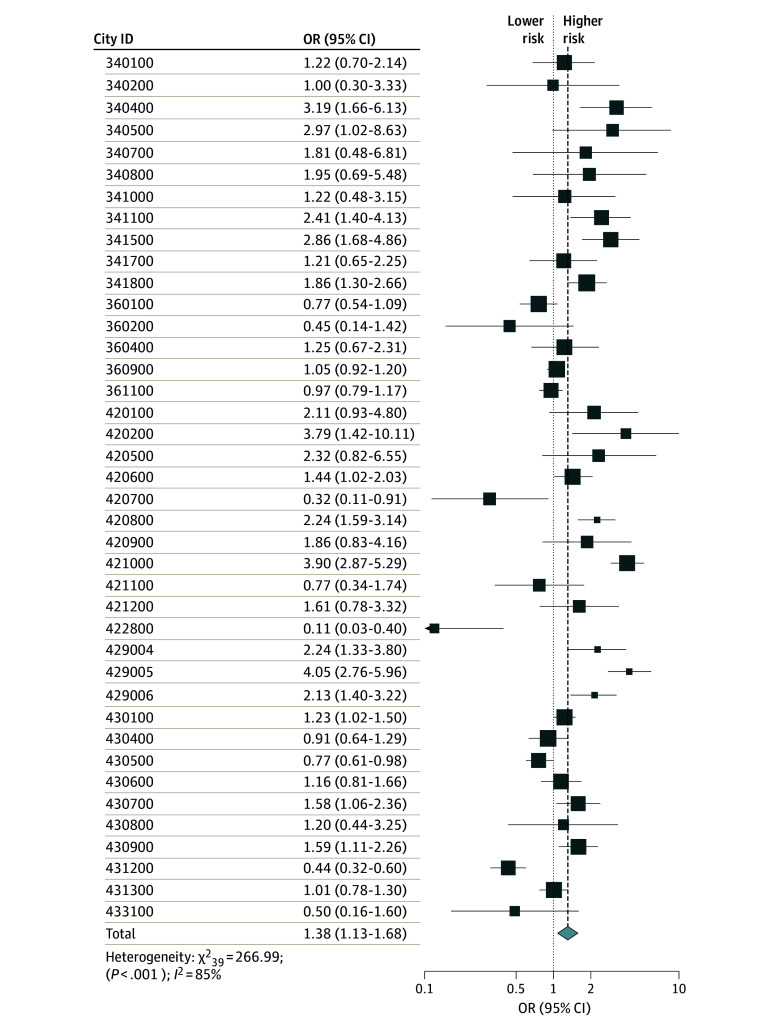
Pooled Risk of Hemorrhagic Fever With Renal Syndrome Within 3 Years After Flooding in Flood Areas The different sizes of the data markers indicate the magnitude of the weight. OR indicates odds ratio.

According to Table 2, random-effect meta-analyses results showed that the risk of all types of HFRS incidence increased in flood areas (OR, 1.38; 95% CI, 1.13-1.68; *P* = .001) (Figure 2; eFigure 10 and eFigure 11 in Supplement 1) but not in nonflood areas (eFigure 12 to eFigure 14 in Supplement 1), with type 1 cases being at highest risk (OR, 1.71; 95% CI, 1.40-2.09; *P* < .001). Furthermore, subgroup analyses revealed that there was a risk of type 1 cases after flooding in Anhui (OR, 2.26; 95% CI, 1.77-2.90; *P* < .001), Hunan (OR, 1.45; 95% CI, 1.09-1.92; *P* = .01), and Hubei Provinces (OR, 2.40; 95% CI, 1.84-3.12; *P* < .001) (eFigure 15 in Supplement 1) but not in Jiangxi Province (OR, 1.09; 95% CI, 0.98-1.21) (Table 2). After flooding, the risk of type 2 cases was only significant in Anhui (OR, 1.90; 95% CI, 1.58-2.32; *P* < .001) and Hubei Provinces (OR, 2.00; 95% CI, 1.45-2.76; *P* < .001). eTable 3 and eTable 4 in Supplement 1 show the results of pairwise comparisons between each subgroup after Bonferroni correction.

**Table 2.** Pooled Effects of the Risk of HFRS After Flooding

Study area	Results of step 1			Results of step 2				
	*I*^2^, %	*Q* value	Exclude^a^	*I*^2^, %	*Q* value	Method	OR (95% CI)	*P* value
**All types of HFRS**								
Nonflood area	66.1	<.001	Shiyan	19.5	.24	FEM	1.06 (0.95-1.18)	.31
Flood area	86.9	<.001	Suizhou	85.4	<.001	REM	1.38 (1.13-1.68)	.001
Subgroup analysis								
Anhui	19.7	.26	NA	NA	NA	FEM	1.90 (1.58-2.32)	<.001
Jiangxi	23.2	.27	NA	NA	NA	FEM	0.99 (0.90-1.11)	.96
Hubei	86.0	<.001	Suizhou	81.8	<.001	REM	2.26 (1.22-4.18)	.009
Hunan	81.6	<.001	Huaihua	61.1	.008	REM	1.10 (0.91-1.33)	.31
**Type 1 HFRS**								
Nonflood area	42.6	.04	Fuzhou	12.6	.31	FEM	1.04 (0.94-1.15)	.47
Flood area^b^	92.0	<.001	Tongling	90.3	<.001	REM	1.71 (1.40-2.09)	<.001
Subgroup analysis								
Anhui	91.6	<.001	Tongling	45.0	.06	REM	2.26 (1.77-2.90)	<.001
Jiangxi	28.0	.24	NA	NA	NA	FEM	1.09 (0.98-1.21)	.10
Hubei	88.8	<.001	Suizhou	75.1	<.001	REM	2.40 (1.84-3.12)	<.001
Hunan	91.0	<.001	Huaihua	83.5	<.001	REM	1.45 (1.09-1.92)	.01
**Type 2 HFRS**								
Nonflood area	19.5	.24	NA	NA	NA	FEM	1.06 (0.95-1.18)	.31
Flood area	85.4	<.001	Jingzhou	82.0	<.001	REM	1.33 (1.10-1.61)	.003
Subgroup analysis								
Anhui	19.7	.26	NA	NA	NA	FEM	1.90 (1.58-2.32)	<.001
Jiangxi	23.2	.27	NA	NA	NA	FEM	1.00 (0.90-1.11)	.96
Hubei	81.8	<.001	Enshi	75.8	<.001	REM	2.00 (1.45-2.76)	<.001
Hunan	81.6	<.001	Huaihua	61.1	.008	REM	1.10 (0.91-1.33)	.31

Abbreviations: FEM, fixed-effect meta-analysis; HFRS, hemorrhagic fever with renal syndrome; NA, not applicable; OR, odds ratio; REM, random-effects meta-analysis.

^a^ Indicates that the city has the highest heterogeneity in step 1, and therefore the variable will be excluded in step 2.

^b^ Due to the low number of type 1 cases in Ezhou and Enshi, these 2 areas are not considered in the pooled effect of the risk of HFRS.

### Associations Between Geographical Factors and the Risk of HFRS After Flooding

The results of the metaregression indicated negative associations between topographic factors (eg, ruggedness index and elevation) and the risk of HFRS (all types) after flooding. However, the distance from rivers was not associated with the risk of type 1 cases after flooding, but the interaction of distance to rivers with the topographic factors was negatively associated with the risk of HFRS, although this interaction was not statistically significant for type 1 cases (Table 3).

**Table 3.** Association of Geographical Factors With the Risk of HFRS After Flooding

Variable	β (95% CI)	*P* value
**All types of HFRS**		
Log (ruggedness index)	−0.22 (−0.35 to −0.01)	.002
Log (elevation)	−0.32 (−0.49 to −0.15)	<.001
Log (distance to rivers)	−0.07 (−0.20 to 0.06)	.27
Log (distance to the Yangtze River)	−0.15 (−0.27 to −0.02)	.02
Log (distance to rivers) × log (ruggedness index)	−0.02 (−0.03 to −0.01)	.003
Log (distance to rivers) × log (elevation)	−0.02 (−0.03 to −0.01)	.002
**Type 1 HFRS**		
Log (ruggedness index)	−0.18 (−0.33 to −0.04)	.01
Log (elevation)	−0.28 (−0.47 to −0.09)	.004
Log (distance to rivers)	0.01 (−0.14 to 0.12)	.91
Log (distance to the Yangtze River)	−0.10 (−0.23 to 0.03)	.12
Log (distance to rivers) × log (ruggedness index)	−0.01 (−0.02 to 0)	.05
Log (distance to rivers) × log (elevation)	−0.01 (−0.03 to 0)	.06
**Type 2 HFRS**		
Log (ruggedness index)	−0.20 (−0.33 to −0.08)	.001
Log (elevation)	−0.29 (−0.46 to −0.12)	<.001
Log (distance to rivers)	−0.07 (−0.19 to 0.06)	.30
Log (distance to the Yangtze River)	−0.13 (−0.25 to 0)	.049
Log (distance to rivers) × log (ruggedness index)	−0.02 (−0.03 to −0.01)	.004
Log (distance to rivers) × log (elevation)	−0.02 (−0.03 to −0.01)	.005

Abbreviation: HFRS, hemorrhagic fever with renal syndrome.

## Discussion

This study found that HFRS cases in the Yangtze River region were mainly distributed in the low-lying areas near rivers and that farmers accounted for 70.6% of the total cases. Although there was a temporary decrease in cases immediately after flooding, the risk of HFRS cases (all types) significantly increased within 3 years, with type 1 cases showing the highest risk. This finding provides needed evidence for developing long-term prevention and control strategies for HFRS after flooding and also offers relevant experience for the prevention of other rodent-borne diseases after floods in other areas. Meta-analysis confirmed the associations, and the risk of type 1 cases increased after flooding in almost all study provinces. In addition, we have adjusted for some concurrent events by evaluating the pooled effect of nonflood areas, to make the results reliable.^33,34^ The metaregression results also showed that topographic factors (eg, elevation and ruggedness index) were negatively associated with the risk of HFRS, while the interaction of the distance to the river with the topographic factors was negatively associated with the risk of HFRS after flooding.

Low-lying areas near water are ideal for human habitation, crop growth, and rodent habitat, which increased the likelihood of rodent-to-rodent and rodent-to-human contact and viral transmission, ultimately leading to the spread of HFRS.^35^ In addition, studies have shown that hantavirus has a longer survival time in moist environments, which increased the risk of pathogen exposure for both humans and hosts.^36,37^ Agricultural areas, often with poor sanitation and the presence of *A agrarius*, can have a further increased HFRS incidence.^28,38^ Given that most farmers are in lower socioeconomic status, providing free vaccination to farmers in high-risk areas is a feasible measure to address social inequities.

Long-term attention should be paid to the prevention and control of HFRS after flooding, with special focus on the high risk of type 1 cases. After floods, *R norvegicus* tend to gather in residential areas and inhabit relatively safe spaces, such as sewers and warehouses.^39^ They reproduce over multiple generations, posing a sustained and serious threat to nearby residents.^20,28^ Measures such as short-term rodent control, reduced crop production, and drowning of wild rats may be associated with the initial decrease in type 2 cases after flooding.^40^ However, there was an increase in type 2 cases within the 3-year period after flooding, likely due to increased rodent-to-rodent contact during flooding and elevated infection levels in their offspring.^20^ In Jiangxi and Hunan provinces, no significant association between floods and type 2 cases was observed, possibly due to the relatively mild levels of flooding in these areas. Therefore, further research is needed to validate the association between floods and the risk of HFRS.

The results of metaregression analyses showed a negative association between elevation or ruggedness index and the risk of HFRS incidence after flooding. In the areas with higher elevation and greater terrain ruggedness, there are more shelters available for rodents and humans after flooding, leading to a decrease in the frequency of rodent-to-human contact and a reduction in the risk of HFRS incidence. Furthermore, the interaction of distance to rivers with the topographic factors was negatively associated with the risk of HFRS after flooding. Low-lying areas near rivers often exhibit high levels of economic development, agricultural production, rodent density, and population density, which could be because areas near rivers and flat terrain do not facilitate flood drainage, resulting in longer periods of rodent aggregation and faster virus spread.^41^ However, no similar associations were found for type 1 cases, which may be due to the fact that type 1 cases are caused mainly by *R norvegicus* living in residential areas. After flooding, *R norvegicus* may tend to move toward shelters in residential areas rather than higher elevations. Therefore, comprehensive HFRS control measures should be implemented in all flood-affected residential areas. In agricultural areas, efforts should be intensified to exterminate rodents in higher safe shelters that are not affected by flooding. In addition, relevant evidence is crucial for enhancing early planning of climate-sensitive disease control measures and flood preparedness, as the world will face more frequent flood events amid future global changes.^42^

### Limitations

This study has some limitations. First, although *R norvegicus* and *A agrarius* are used to explain type 1 and type 2 cases, the surveillance system does not record which rodent species infected the patients as a natural reservoir, simply because of the high prevalence of HFRS caused by these 2 rodent species during the corresponding seasons. Second, although interrupted time series analysis can mitigate the influence of some confounding factors, studies still face challenges such as selection bias and information bias. Therefore, caution is needed when extrapolating the results. Third, short-term measures for rodent control were implemented after flooding, which could have reduced the association of floods with HFRS. Fourth, future studies should focus on conducting more fine-scale analyses of rodent activity patterns after flooding to better understand and explain the risk of HFRS.

## Conclusions

This cross-sectional study suggests that long-term control measures of HFRS (especially type 1 cases) after floods should be implemented, rather than focusing on solely short-term measures. In addition, special attention should be given to assessing the HFRS risk in areas closer to rivers with lower elevations and flatter terrain. These findings can guide public health authorities and other relevant decision-makers in implementing targeted and sustained measures to minimize the effect of HFRS outbreaks in flood areas.

## Notes

Supplement 1. **eMethods.** Definition of HFRS
eReferences.
**eTable 1.** Administrative Codes for Cities in Anhui, Jiangxi, Hubei, Hunan Provinces **eTable 2.** Characteristics of HFRS Cases Before and After Flooding **eTable 3.** Comparison of Different Types of the Pooled Effects of HFRS Risk After Bonferroni Correction in Flood Areas **eTable 4.** Comparison of Different Provinces of the Pooled Effects of HFRS Risk After Bonferroni Correction in Flood Areas **eFigure 1.** Conceptual Model for the Increased Long-Term Risk of HFRS After Flooding **eFigure 2.** The Flood Situation in the Study Area **eFigure 3.** Sample Inclusion and Exclusion Criteria **eFigure 4.** The Geographical Characteristics of the Study Area **eFigure 5.** Temporal Trends in HFRS Incidence in Area Affected by Flooding Compared to Non-Flood Area **eFigure 6.** Temporal Trends in HFRS Incidence in 4 Provinces **eFigure 7.** Diagnostics Results of the ITSA Models **eFigure 8.** The Risk of HFRS Incidence in Various Cities After the Severe Flood **eFigure 9.** Sensitivity Analysis Results of Generalized Additive Models **eFigure 10.** The Pooled Effect of Type 1 Case Risk Within 3 Years After Flooding **eFigure 11.** The Pooled Effect of Type 2 Case Risk Within 3 Years After Flooding **eFigure 12.** The Pooled Effect of HFRS Risk Within 3 Years for all Types in Non-Flood Areas **eFigure 13.** The Pooled Effect of Type 1 Case Risk Within 3 Years in Non-Flood Areas **eFigure 14.** The Pooled Effect of Type 2 Case Risk Within 3 Years in Non-Flood Areas **eFigure 15.** Subgroup Analyses of the Pooled Effects of HFRS Risk Within 3 Years for all Types in Flood Areas

Supplement 2.
Data Sharing Statement
