# Supplementary material for: The 2016 Severe Floods and Incidence of Hemorrhagic Fever With Renal Syndrome in the Yangtze River Basin
Source: JAMA Netw Open. 2024 Aug 22;7(8):e2429682. doi: 10.1001/jamanetworkopen.2024.29682 (PMC11342140; doi:10.1001/jamanetworkopen.2024.29682)
Supplement: Supplement 1. — eMethods. Definition of HFRS eReferences. eTable 1. Administrative Codes for Cities in Anhui, Jiangxi, Hubei, Hunan Provinces eTable 2. Characteristics of HFRS Cases Before and After Flooding eTable 3. Comparison of Different Types of the Pooled Effects of HFRS Risk After Bonferroni Correction in Flood Areas eTable 4. Comparison of Different Provinces of the Pooled Effects of HFRS Risk After Bonferroni Correction in Flood Areas eFigure 1. Conceptual Model for the Increased Long-Term Risk of HFRS After Flooding eFigure 2. The Flood Situation in the Study Area eFigure 3. Sample Inclusion and Exclusion Criteria eFigure 4. The Geographical Characteristics of the Study Area eFigure 5. Temporal Trends in HFRS Incidence in Area Affected by Flooding Compared to Non-Flood Area eFigure 6. Temporal Trends in HFRS Incidence in 4 Provinces eFigure 7. Diagnostics Results of the ITSA Models eFigure 8. The Risk of HFRS Incidence in Various Cities After the Severe Flood eFigure 9. Sensitivity Analysis Results of Generalized Additive Models eFigure 10. The Pooled Effect of Type 1 Case Risk Within 3 Years After Flooding eFigure 11. The Pooled Effect of Type 2 Case Risk Within 3 Years After Flooding eFigure 12. The Pooled Effect of HFRS Risk Within 3 Years for all Types in Non-Flood Areas eFigure 13. The Pooled Effect of Type 1 Case Risk Within 3 Years in Non-Flood Areas eFigure 14. The Pooled Effect of Type 2 Case Risk Within 3 Years in Non-Flood Areas eFigure 15. Subgroup Analyses of the Pooled Effects of HFRS Risk Within 3 Years for all Types in Flood Areas [file jamanetwopen-e2429682-s001.pdf]

Supplementary Online Content

Ji H, Li K, Shang M, Wang Z, Liu Q. The 2016 severe floods and incidence of hemorrhagic fever with renal syndrome in the Yangtze River basin. *JAMA Netw Open.* 2024;7(8):e2429682. doi:10.1001/jamanetworkopen.2024.29682

eMethods. Definition of HFRS
eReferences.
eTable 1. Administrative Codes for Cities in Anhui, Jiangxi, Hubei, Hunan Provinces
eTable 2. Characteristics of HFRS Cases Before and After Flooding
eTable 3. Comparison of Different Types of the Pooled Effects of HFRS Risk After Bonferroni Correction in Flood Areas
eTable 4. Comparison of Different Provinces of the Pooled Effects of HFRS Risk After Bonferroni Correction in Flood Areas
eFigure 1. Conceptual Model for the Increased Long-Term Risk of HFRS After Flooding
eFigure 2. The Flood Situation in the Study Area
eFigure 3. Sample Inclusion and Exclusion Criteria
eFigure 4. The Geographical Characteristics of the Study Area
eFigure 5. Temporal Trends in HFRS Incidence in Area Affected by Flooding Compared to Non-Flood Area
eFigure 6. Temporal Trends in HFRS Incidence in 4 Provinces
eFigure 7. Diagnostics Results of the ITSA Models
eFigure 8. The Risk of HFRS Incidence in Various Cities After the Severe Flood
eFigure 9. Sensitivity Analysis Results of Generalized Additive Models
eFigure 10. The Pooled Effect of Type 1 Case Risk Within 3 Years After Flooding
eFigure 11. The Pooled Effect of Type 2 Case Risk Within 3 Years After Flooding
eFigure 12. The Pooled Effect of HFRS Risk Within 3 Years for all Types in Non-Flood Areas
eFigure 13. The Pooled Effect of Type 1 Case Risk Within 3 Years in Non-Flood Areas
eFigure 14. The Pooled Effect of Type 2 Case Risk Within 3 Years in Non-Flood Areas
eFigure 15. Subgroup Analyses of the Pooled Effects of HFRS Risk Within 3 Years for all Types in Flood Areas

This supplementary material has been provided by the authors to give readers additional information about their work.

© 2024 Ji H et al. *JAMA Network Open.*

eMethods. Definition of HFRS

HFRS cases are defined as individuals who have a history of living in HFRS-endemic areas or have had contact with rodent feces, saliva, and urine within 2 months before the onset of the disease. Clinical manifestations include fever, chills, bleeding, headache, back pain, abdominal pain, acute renal failure, and hypotension. In addition, patients must meet at least one laboratory diagnostic criterion: positive for HTNV-specific immunoglobulin M, a four-fold increase in HTNV-specific immunoglobulin G titers, positive detection of HTNV-specific ribonucleic acid by reverse transcription-polymerase chain reaction in clinical specimens, or isolation of HTNV2 from clinical specimens[1,2].

© 2024 Ji H et al. *JAMA Network Open.*

eReferences.

1. Wang Y, Wei X, Jia R, et al. The Spatiotemporal Pattern and Its Determinants of Hemorrhagic Fever With Renal Syndrome in Northeastern China: Spatiotemporal Analysis. *JMIR Public Health Surveill*. 2023;9:e42673. doi:10.2196/42673

2. National Health Commission of the People's Republic of China. Diagnostic criteria for epidemic hemorrhagic fever. Published online 2021.

eTable 1. Administrative Codes for Cities in Anhui, Jiangxi, Hubei, Hunan Provinces

eTable 1. Administrative codes for cities in Anhui, Jiangxi, Hubei, Hunan provinces.

Anhui		Jiangxi		Hubei		Hunan	
City name	Code	City name	Code	City name	Code	City name	Code
Hefei	340100	Nanchang	360100	Wuhan	420100	Changsha	430100
Wuhu	340200	Jingdezhen	360200	Huangshi	420200	Zhuzhou	430200
Bengbu	340300	Pingxiang	360300	Shiyan	420300	Xiangtan	430300
Huainan	340400	Jiujiang	360400	Yichang	420500	Hengyang	430400
Maanshan	340500	Xinyu	360500	Xiangyang	420600	Shaoyang	430500
Huaibei	340600	Yingtan	360600	Ezhou	420700	Yueyang	430600
Tongling	340700	Ganzhou	360700	Jingmen	420800	Changde	430700
Anqing	340800	Jian	360800	Xiaogan	420900	Zhangjiajie	430800
Huangshan	341000	Yichun	360900	Jingzhou	421000	Yiyang	430900
Chuzhou	341100	Fuzhou	361000	Huanggang	421100	Chenzhou	431000
Fuyang	341200	Shangrao	361100	Xianning	421200	Yongzhou	431100
Suzhou	341300			Suizhou	421300	Huaihua	431200
Liuan	341500			Enshitujiazumiaozu	422800	Loudi	431300
Bozhou	341600			Xiantao	429004	Xiangxitujiazumiaozu	433100
Chizhou	341700			Qianjiang	429005		
Xuancheng	341800			Tianmen	429006		
				Shennongjialin	429021		

© 2024 Ji H et al. *JAMA Network Open.*

eTable 2. Characteristics of HFRS Cases Before and After Flooding

eTable 2. Characteristics of HFRS cases before and after flooding.

Characteristics	Groups	Pre-flood	Post-flood	P value
All cases		5216	6529	
Age (mean (SD))		47.14 (16.20)	49.75 (15.80)	<0.001
Diagnosis time (median [IQR])		6.00 [3.00, 10.00]	5.00 [2.00, 10.00]	<0.001
Gender (%)	Women	1479 (28.4)	1857 (28.4)	0.933
	Men	3737 (71.6)	4672 (71.6)	
Provinces (%)	Anhui	500 (9.6)	831 (12.7)	<0.001
	Jiangxi	1931 (37.0)	1943 (29.8)	
	Hubei	746 (14.3)	1795 (27.5)	
	Hunan	2039 (39.1)	1960 (30.0)	
Occupation (%)	Others[a]	1573 (30.2)	1881 (28.8)	0.116
	Farmers	3643 (69.8)	4648 (71.2)	
HFRS types (%)	Type 1	2193 (42.0)	3129 (47.9)	<0.001
	Type 2	3023 (58.0)	3400 (52.1)	
Death (%)	No	5174 (99.2)	6483 (99.3)	0.602
	Yes	42 (0.8)	46 (0.7)	
Flood area (%)	Non-flood area	1051 (20.1)	1087 (16.6)	<0.001
	Flood area	4165 (79.9)	5442 (83.4)	
Patient location (%)	Native county	2411 (46.2)	3098 (47.4)	0.118
	Native city	1376 (26.4)	1603 (24.6)	
	Native Province	1163 (22.3)	1509 (23.1)	
	Others[b]	266 (5.1)	319 (4.9)	

Note: [a] represented occupations other than farmers, including civil servants, corporate employees, business operators, housewives, etc.; [b] represented individuals from other provinces or regions (including Hong Kong, Macao, Taiwan) or non-Chinese nationals.

© 2024 Ji H et al. *JAMA Network Open.*

eTable 3. Comparison of Different Types of the Pooled Effects of HFRS Risk After Bonferroni Correction in Flood Areas

eTable 3. Comparison of different types of the pooled effects of HFRS risk after Bonferroni correction in flood areas

	Significant after Bonferroni correction	
Comparison	P value	Significant or not
All Cities: Type1 vs Type2	<0.001	Ture
Hubei: Type1 vs Type2	0.047	Ture
Anhui: Type1 vs Type2	0.545	Flase
Hunan: Type1 vs Type2	0.008	Ture
Jiangxi: Type1 vs Type2	0.238	Flase

© 2024 Ji H et al. *JAMA Network Open.*

eTable 4. Comparison of Different Provinces of the Pooled Effects of HFRS Risk After Bonferroni Correction in Flood Areas

eTable 4. Comparison of different provinces of the pooled effects of HFRS risk after Bonferroni correction in flood areas

Comparison	Significant after Bonferroni correction		
	P value (All types)	P value (Type 1)	P value (Type 2)
Anhui VS Hubei	0.179 [a]	0.159 [a]	0.835 [a]
Anhui VS Hunan	<0.001 [b]	<0.001 [b]	<0.001 [b]
Anhui VS Jiangxi	<0.001 [b]	<0.001 [b]	<0.001 [b]
Hubei VS Hunan	<0.001 [b]	<0.001 [b]	<0.001 [b]
Hubei VS Jiangxi	<0.001 [b]	<0.001 [b]	<0.001 [b]
Hunan VS Jiangxi	0.975 [a]	0.197 [a]	0.975 [a]

Note: a means the difference was not statistically significant after Bonferroni correction; b means the difference was statistically significant after Bonferroni correction.

© 2024 Ji H et al. *JAMA Network Open.*

eFigure 1. Conceptual Model for the Increased Long-Term Risk of HFRS After Flooding

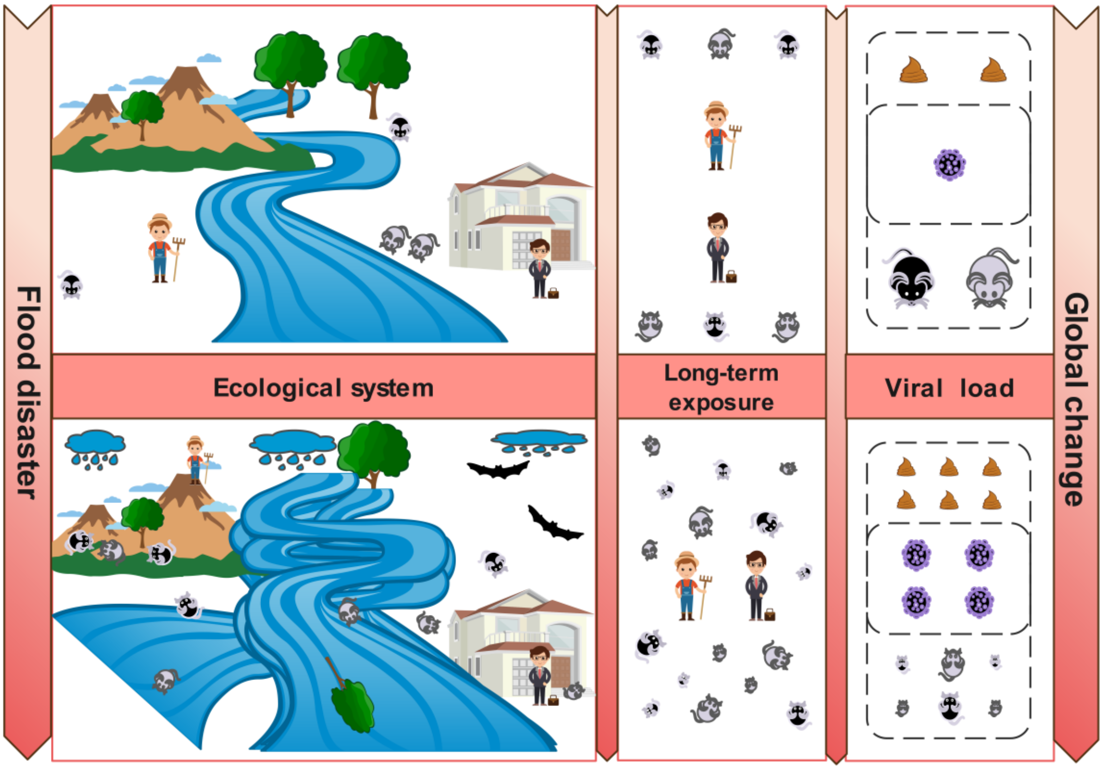

eFigure 1. Conceptual model for the increased long-term risk of HFRS after flooding.

© 2024 Ji H et al. *JAMA Network Open.*

eFigure 2. The Flood Situation in the Study Area

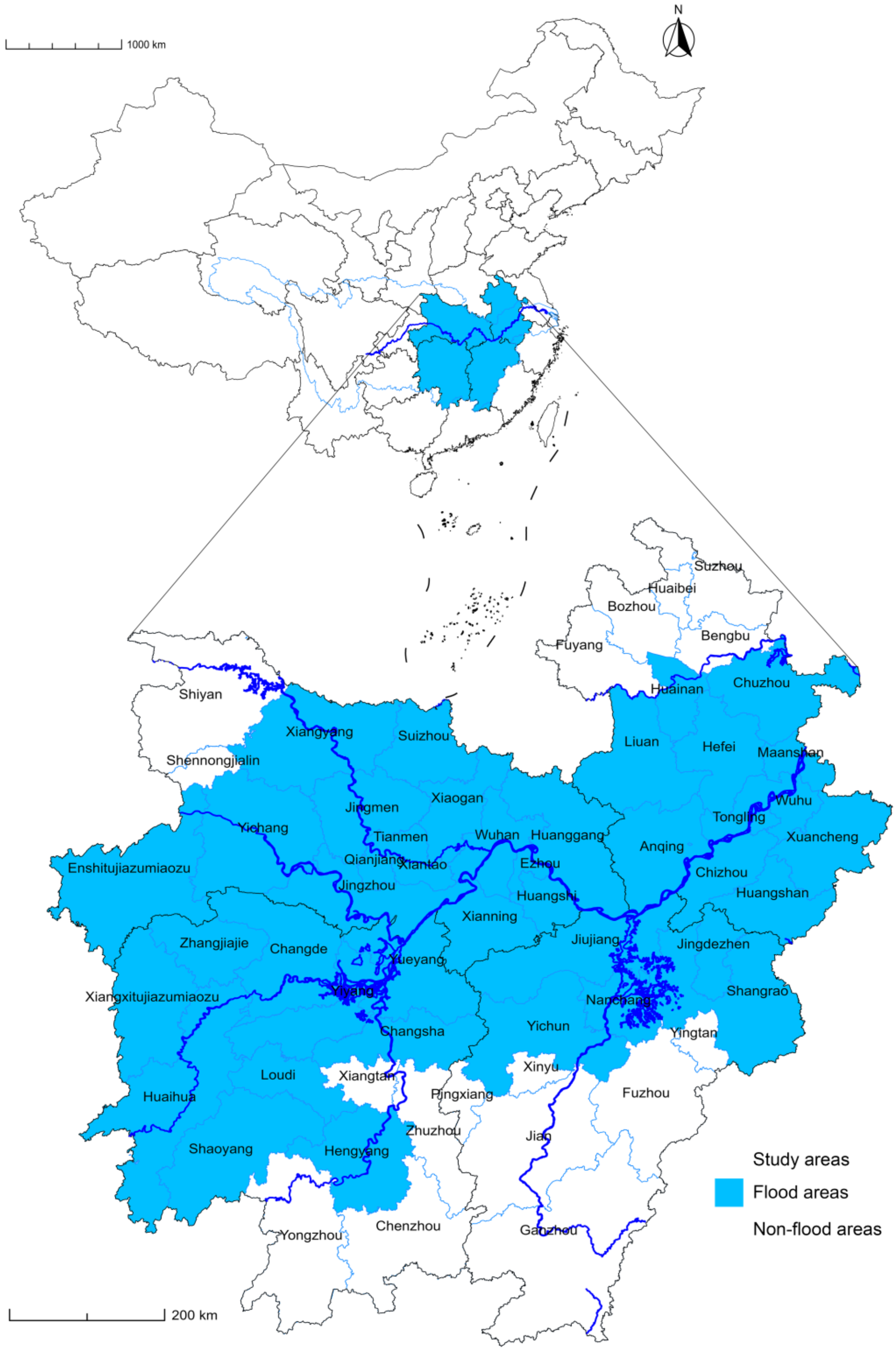

eFigure 2. The flood situation in the study area (The study area has a latitude range of 34°38' N to 119°37' N and a longitude range of 108°21' E to 119°37' E).

© 2024 Ji H et al. *JAMA Network Open.*

eFigure 3. Sample Inclusion and Exclusion Criteria

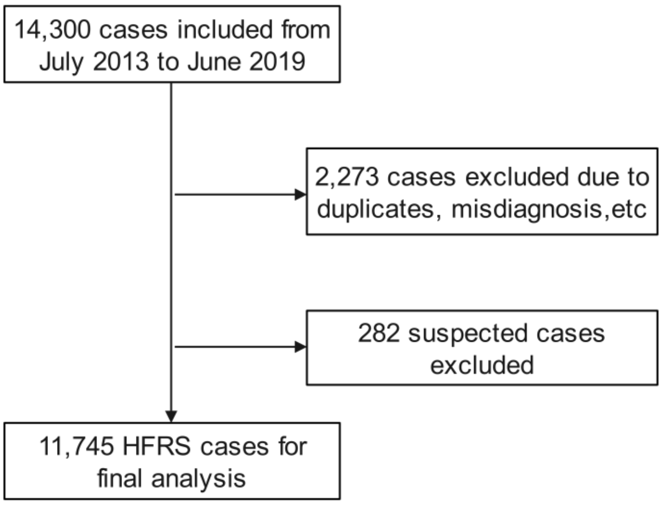

eFigure 3. Sample inclusion and exclusion criteria.

© 2024 Ji H et al. *JAMA Network Open.*

eFigure 4. The Geographical Characteristics of the Study Area

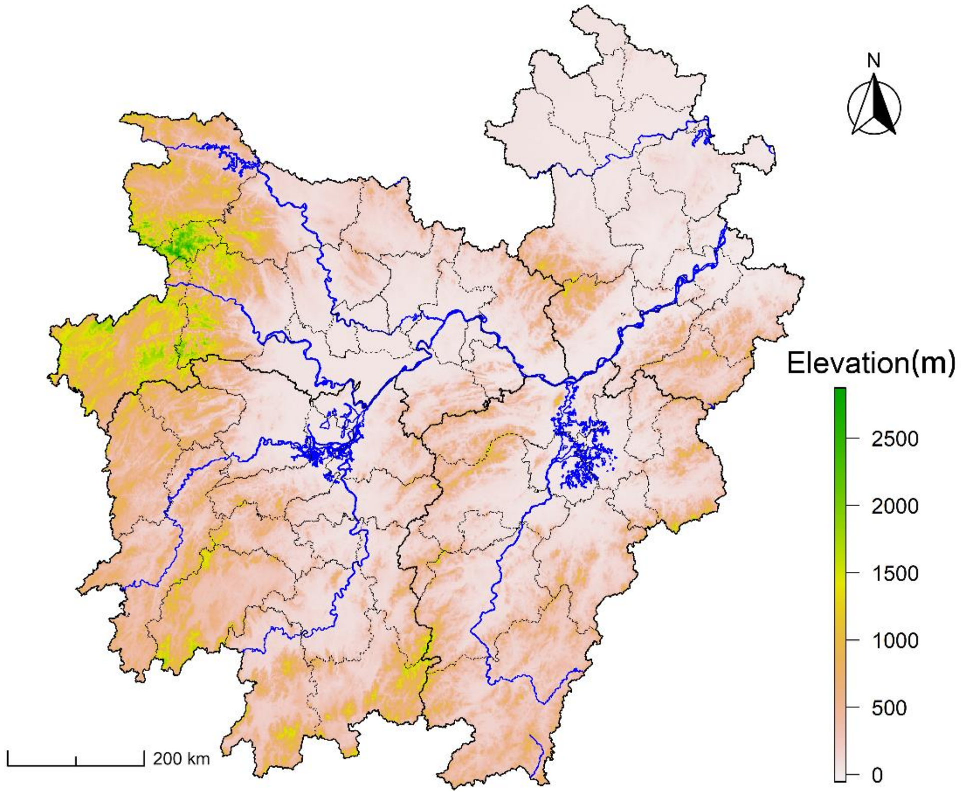

eFigure 4. The geographical characteristics of the study area.

© 2024 Ji H et al. *JAMA Network Open.*

eFigure 5. Temporal Trends in HFRS Incidence in Area Affected by Flooding Compared to Non-Flood Area

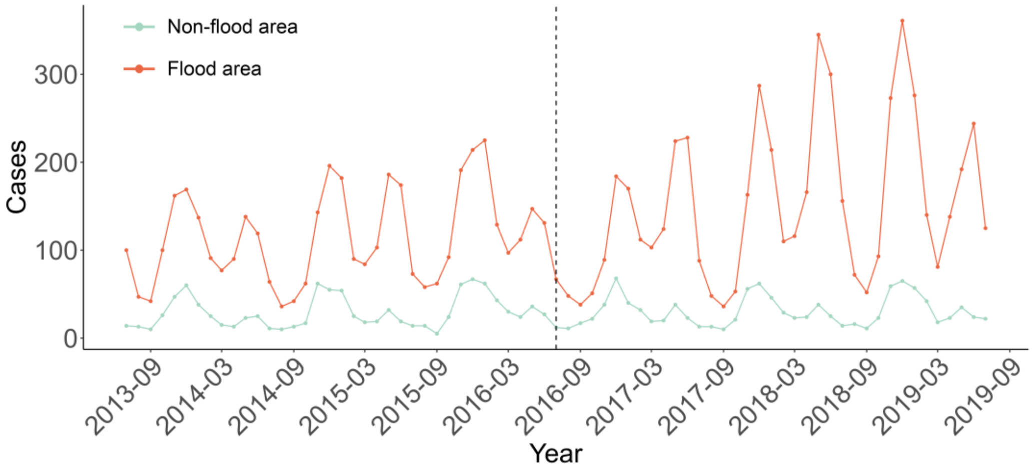

eFigure 5. Temporal trends in HFRS incidence in areas affected by floods compared to non-flood areas.

© 2024 Ji H et al. *JAMA Network Open.*

eFigure 6. Temporal Trends in HFRS Incidence in 4 Provinces

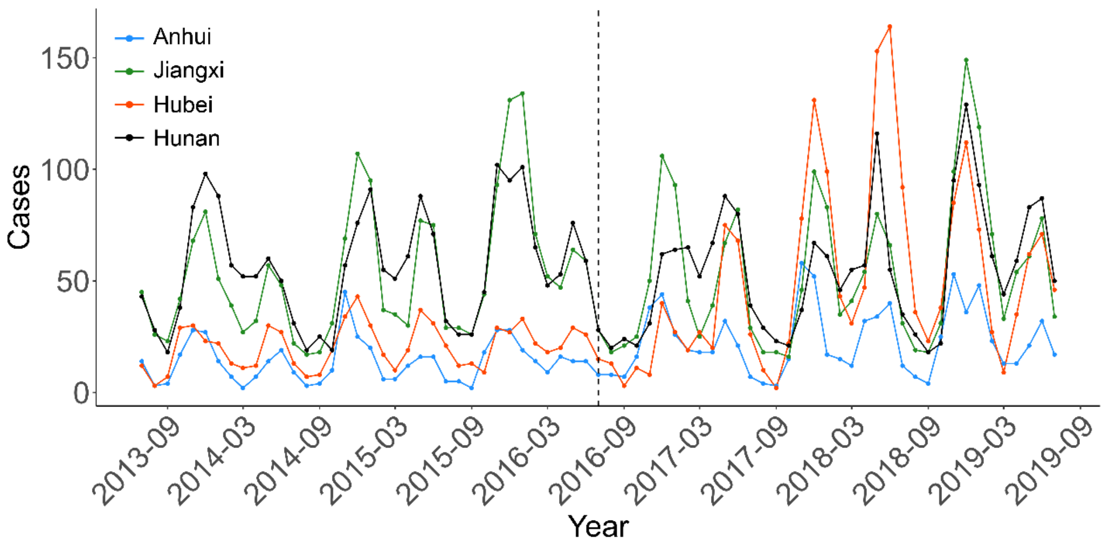

eFigure 6. Temporal trends in HFRS incidence in 4 provinces.

© 2024 Ji H et al. *JAMA Network Open.*

eFigure 7. Diagnostics Results of the ITSA Models

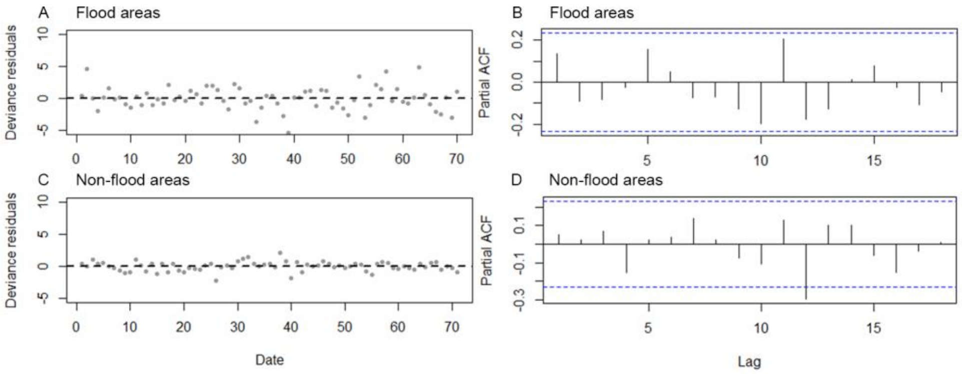

eFigure 7. Diagnostics results of the ITSA models.

© 2024 Ji H et al. *JAMA Network Open.*

eFigure 8. The Risk of HFRS Incidence in Various Cities After the Severe Flood

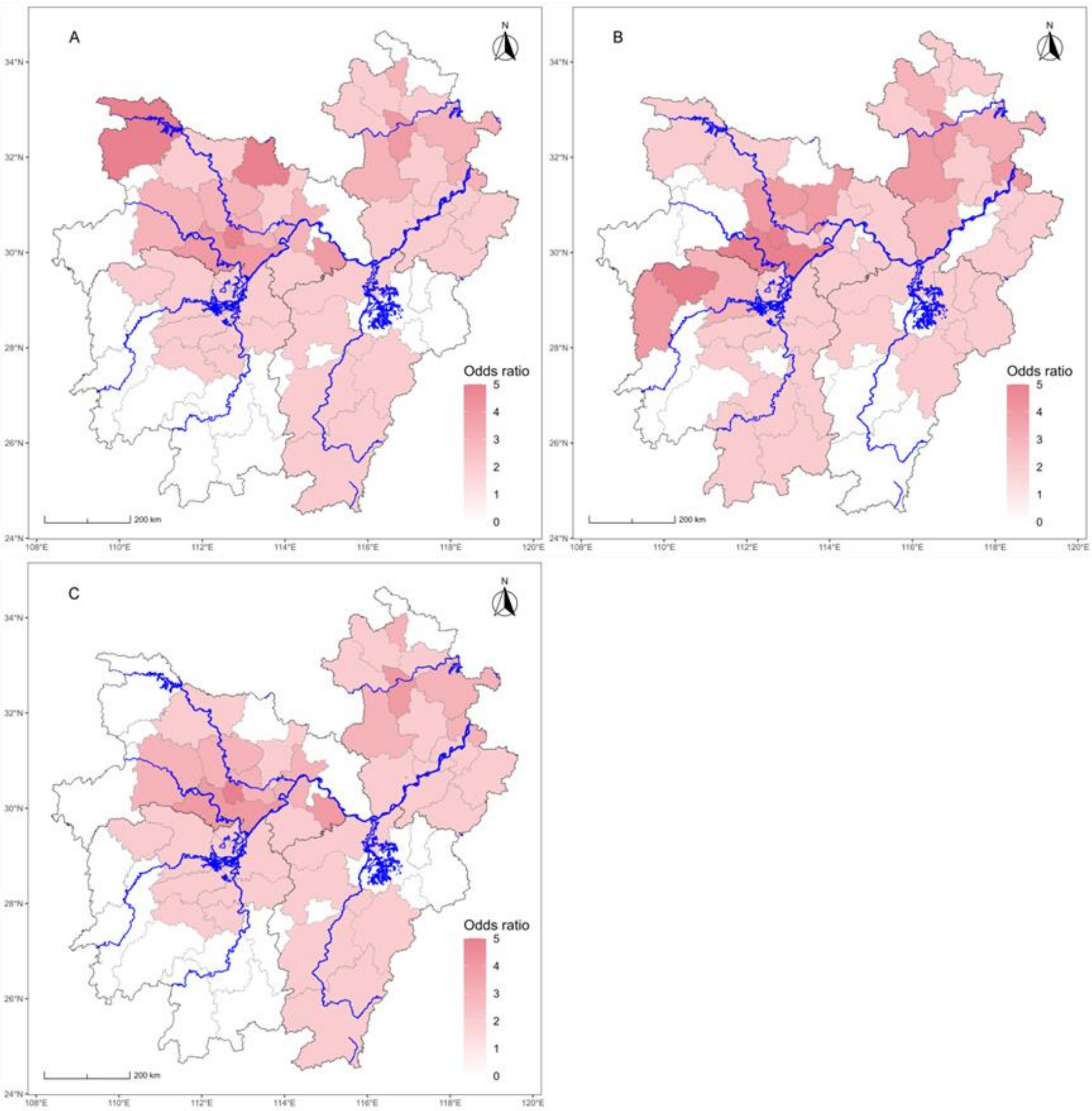

eFigure 8. The risk of HFRS incidence in various cities after the severe flood (A: All types cases; B: Type 1 cases; C: Type 2 cases).

© 2024 Ji H et al. *JAMA Network Open.*

eFigure 9. Sensitivity Analysis Results of Generalized Additive Models

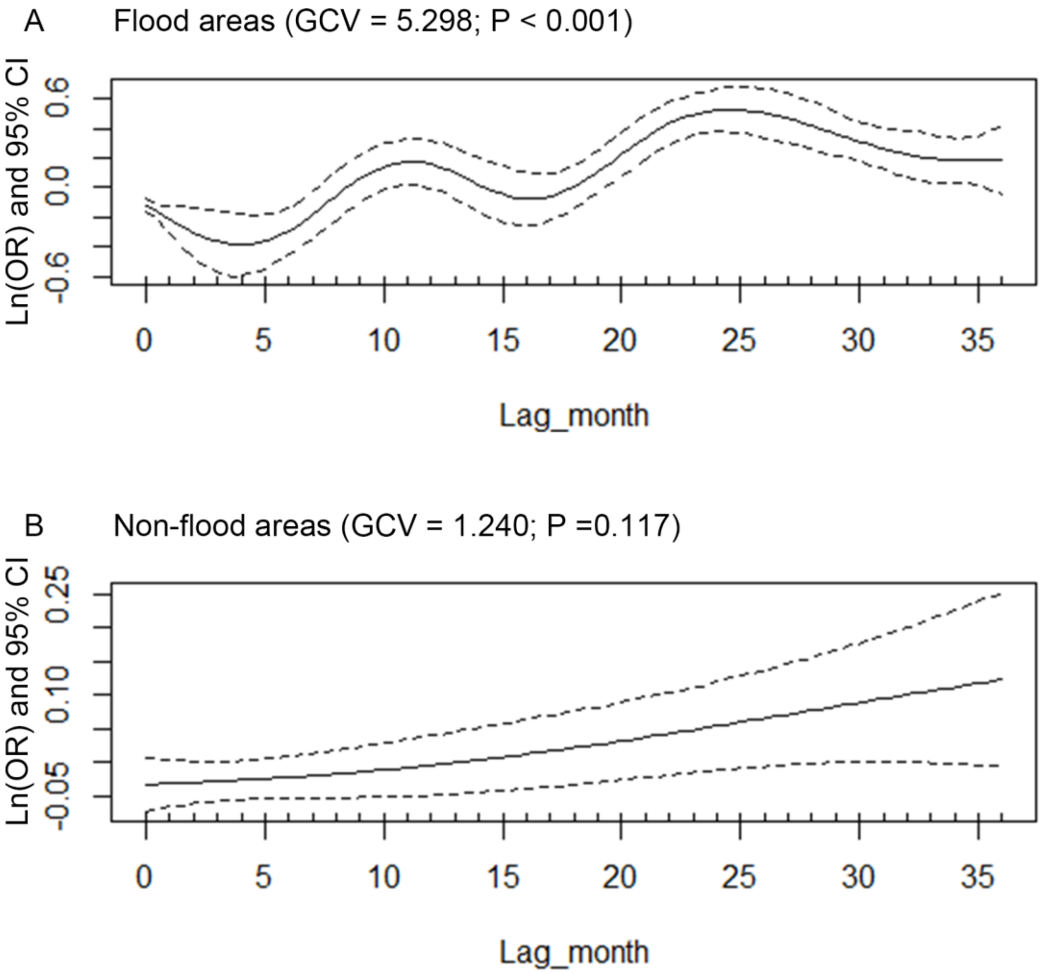

eFigure 9. Sensitivity analysis results of generalized additive models.

© 2024 Ji H et al. *JAMA Network Open.*

eFigure 10. The Pooled Effect of Type 1 Case Risk Within 3 Years After Flooding

Source	OR (95% CI)
340100	1.73 [1.02; 2.93]
340200	1.92 [0.91; 4.06]
340400	3.87 [1.69; 8.84]
340500	3.29 [1.31; 8.28]
340800	2.68 [1.22; 5.89]
341000	1.92 [0.85; 4.36]
341100	2.83 [1.90; 4.22]
341500	3.44 [2.22; 5.32]
341700	0.99 [0.54; 1.82]
341800	2.00 [1.51; 2.63]
360100	0.90 [0.67; 1.21]
360200	1.46 [0.65; 3.30]
360400	1.57 [0.95; 2.62]
360900	1.04 [0.91; 1.19]
361100	1.21 [0.99; 1.49]
420100	1.95 [1.00; 3.80]
420200	1.79 [0.91; 3.51]
420500	0.84 [0.39; 1.80]
420600	1.83 [1.35; 2.48]
420800	3.35 [2.45; 4.59]
420900	3.69 [1.59; 8.58]
421000	4.01 [3.10; 5.20]
421100	1.79 [0.87; 3.66]
421200	1.85 [0.90; 3.83]
421300	0.36 [0.22; 0.58]
429004	1.71 [1.19; 2.47]
429005	4.52 [3.07; 6.65]
429006	2.85 [1.98; 4.10]
430100	1.36 [1.14; 1.63]
430400	1.01 [0.74; 1.37]
430500	0.76 [0.61; 0.95]
430600	1.44 [1.03; 2.00]
430700	2.44 [1.74; 3.42]
430800	4.21 [1.18; 14.96]
430900	1.47 [1.21; 1.78]
431200	0.40 [0.30; 0.55]
431300	1.24 [1.03; 1.49]
433100	3.91 [1.52; 10.06]
Total	1.71 [1.40; 2.09]

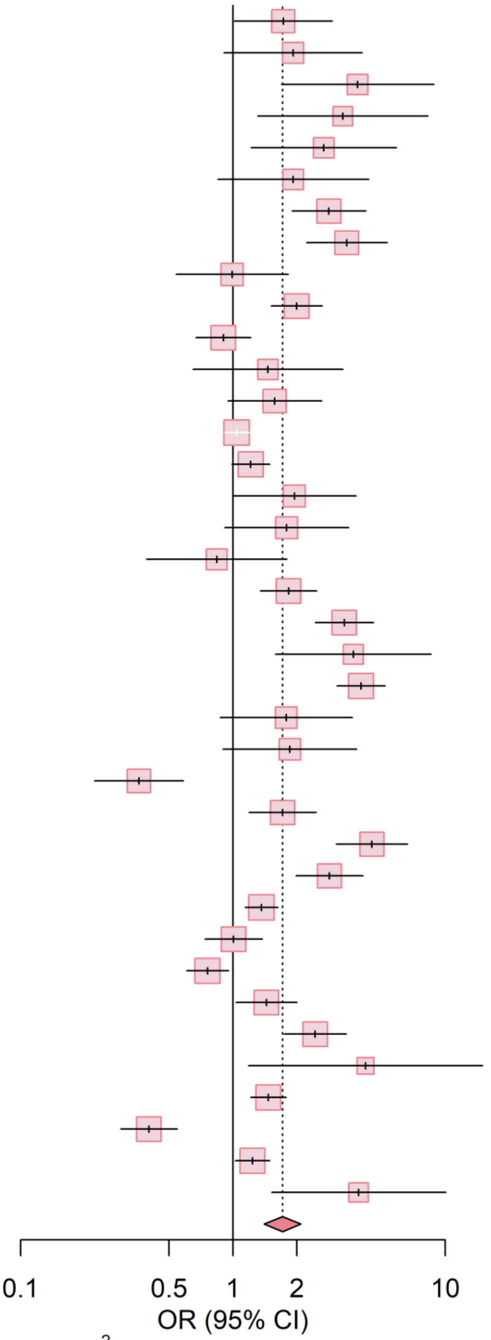

Heterogeneity: $\chi^2_{37} = 379.80$ ($P < .001$), $I^2 = 90\%$

eFigure 10. The pooled effect of type 1 case risk within 3 years after flooding (result of random effect model).

© 2024 Ji H et al. *JAMA Network Open.*

eFigure 11. The Pooled Effect of Type 2 Case Risk Within 3 Years After Flooding

Source	OR (95% CI)
340100	1.22 [0.70; 2.14]
340200	1.00 [0.30; 3.33]
340400	3.19 [1.66; 6.13]
340500	2.97 [1.02; 8.63]
340700	1.81 [0.48; 6.81]
340800	1.95 [0.69; 5.48]
341000	1.22 [0.48; 3.15]
341100	2.41 [1.40; 4.13]
341500	2.86 [1.68; 4.86]
341700	1.21 [0.65; 2.25]
341800	1.86 [1.30; 2.66]
360100	0.77 [0.54; 1.09]
360200	0.45 [0.14; 1.42]
360400	1.25 [0.67; 2.31]
360900	1.05 [0.92; 1.20]
361100	0.97 [0.79; 1.17]
420100	2.11 [0.93; 4.80]
420200	3.79 [1.42; 10.11]
420500	2.32 [0.82; 6.55]
420600	1.44 [1.02; 2.03]
420700	0.32 [0.11; 0.91]
420800	2.24 [1.59; 3.14]
420900	1.86 [0.83; 4.16]
421100	0.77 [0.34; 1.74]
421200	1.61 [0.78; 3.32]
422800	0.11 [0.03; 0.40]
429004	2.24 [1.33; 3.80]
429005	4.05 [2.76; 5.96]
429006	2.13 [1.40; 3.22]
430100	1.23 [1.02; 1.50]
430400	0.91 [0.64; 1.29]
430500	0.77 [0.61; 0.98]
430600	1.16 [0.81; 1.66]
430700	1.58 [1.06; 2.36]
430800	1.20 [0.44; 3.25]
430900	1.59 [1.11; 2.26]
431200	0.44 [0.32; 0.60]
431300	1.01 [0.78; 1.30]
433100	0.50 [0.16; 1.60]
Total	1.33 [1.10; 1.61]

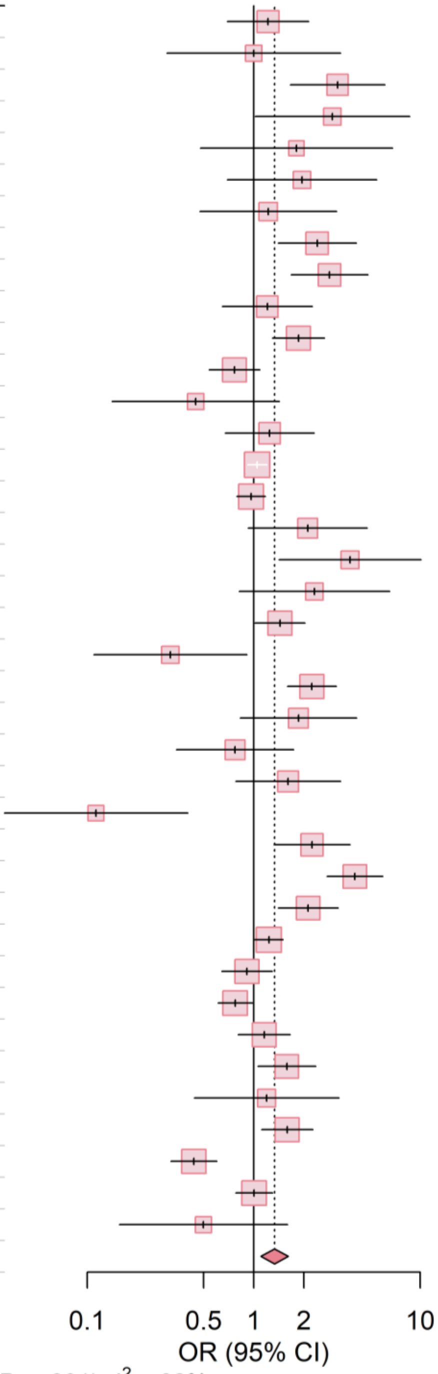

Heterogeneity: $\chi^2_{38} = 211.35$ ($P < .001$), $I^2 = 82\%$

eFigure 11. The pooled effect of type 2 case risk within 3 years after flooding (result of random effect models).

© 2024 Ji H et al. *JAMA Network Open.*

eFigure 12. The Pooled Effect of HFRS Risk Within 3 Years for all Types in Non-Flood Areas

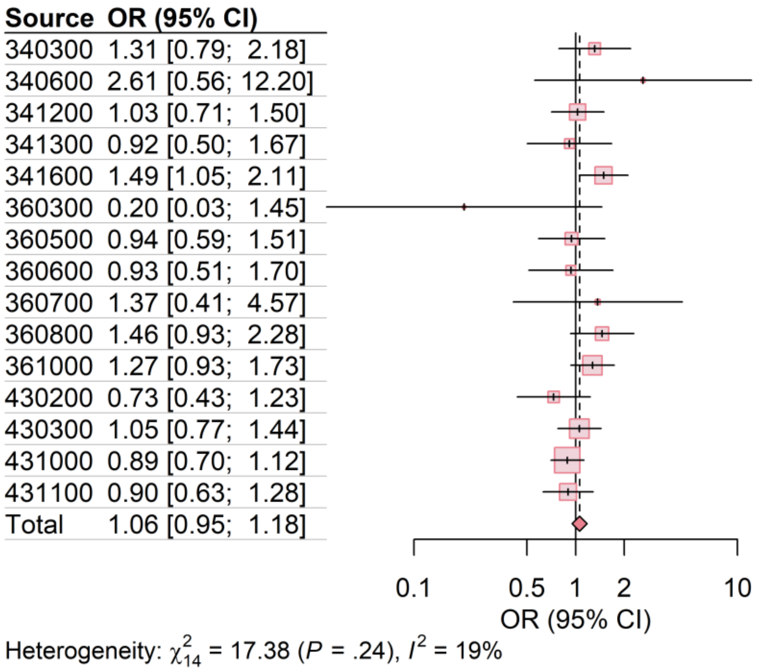

eFigure 12. The pooled effect of HFRS risk within 3 years for all types in non-flood areas (result of fixed effect model).

© 2024 Ji H et al. *JAMA Network Open.*

eFigure 13. The Pooled Effect of Type 1 Case Risk Within 3 Years in Non-Flood Areas

Source	OR (95% CI)
340300	0.85 [0.52; 1.37]
340600	1.00 [0.59; 1.70]
341200	1.04 [0.72; 1.51]
341300	1.21 [0.64; 2.27]
341600	2.10 [1.20; 3.67]
360300	0.61 [0.22; 1.67]
360500	0.81 [0.58; 1.15]
360600	1.17 [0.72; 1.89]
360700	0.66 [0.19; 2.31]
360800	0.73 [0.44; 1.20]
420300	1.00 [0.59; 1.70]
430200	1.23 [0.75; 2.02]
430300	0.99 [0.81; 1.22]
431000	1.03 [0.83; 1.28]
431100	1.33 [0.99; 1.80]
Total	1.04 [0.94; 1.14]

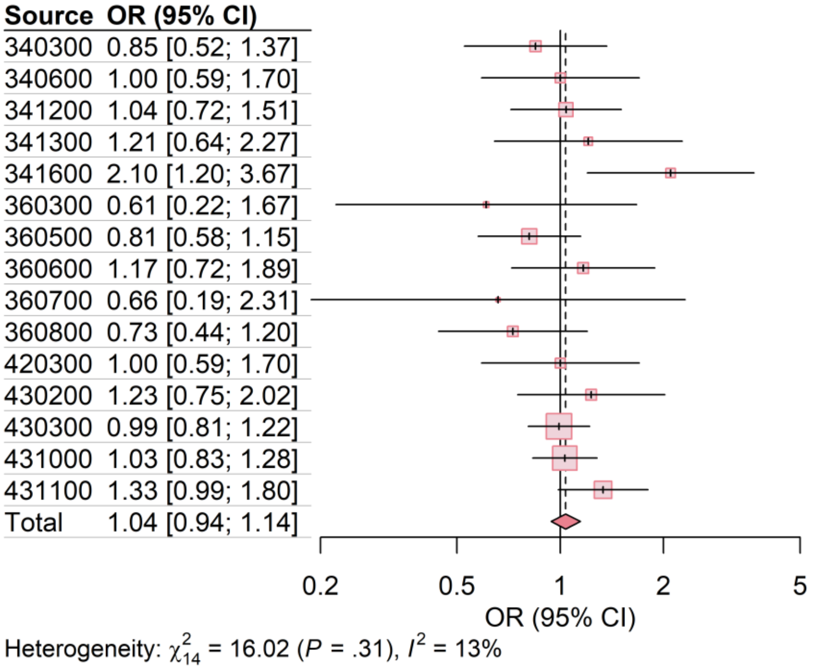

Heterogeneity: χ^2_{14} = 16.02 (*P* = .31), I^2 = 13%

eFigure 13. The pooled effect of type 1 case risk within 3 years in non-flood areas (result of fixed effect model).

© 2024 Ji H et al. *JAMA Network Open.*

eFigure 14. The Pooled Effect of Type 2 Case Risk Within 3 Years in Non-Flood Areas

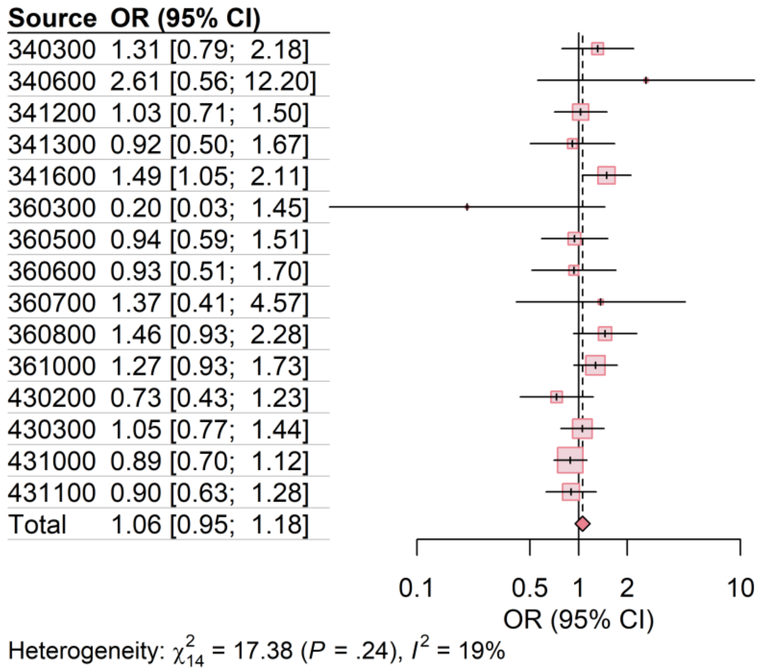

eFigure 14. The pooled effect of type 2 case risk within 3 years in non-flood areas (result of fixed effect model).

© 2024 Ji H et al. *JAMA Network Open.*

eFigure 15. Subgroup Analyses of the Pooled Effects of HFRS Risk Within 3 Years for all Types in Flood Areas

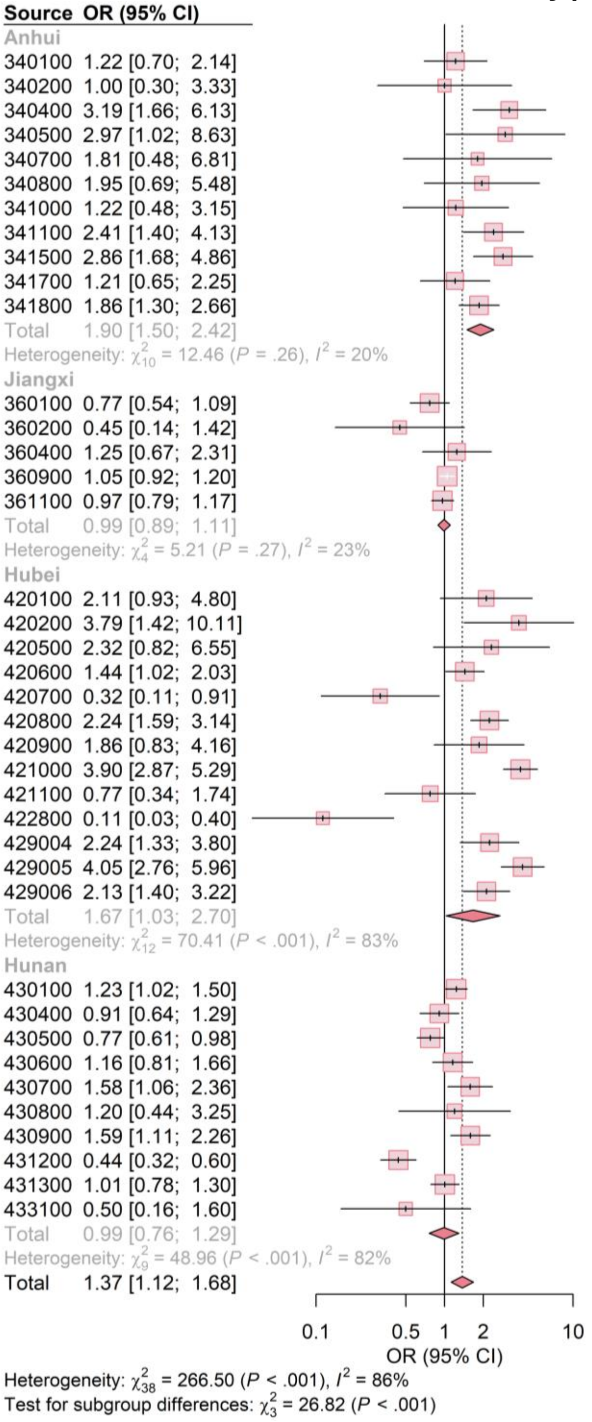

eFigure 15. Subgroup analyses of the pooled effects of HFRS risk within 3 years for all types in flood areas (result of random effect models).

© 2024 Ji H et al. *JAMA Network Open.*
