# Supplementary material for: The 2016 Severe Floods and Incidence of Hemorrhagic Fever With Renal Syndrome in the Yangtze River Basin
Source: JAMA Netw Open. 2024 Aug 22;7(8):e2429682. doi: 10.1001/jamanetworkopen.2024.29682 (PMC11342140; doi:10.1001/jamanetworkopen.2024.29682)
Supplement: Supplement 2. — Data Sharing Statement [file jamanetwopen-e2429682-s002.pdf]

Data Sharing Statement

Ji. The 2016 Severe Floods and Incidence of Hemorrhagic Fever With Renal Syndrome in the Yangtze River Basin. *JAMA Netw Open*. Published August 22, 2024.
doi:10.1001/jamanetworkopen.2024.29682

Data
Data available: No

Additional Information
Explanation for why data not available: The HFRS data underlying the results presented in the study cannot be shared publicly because of the limitation of data availability in the data management rule of Chinese Center for Disease Control and Prevention. Access to these data may be requested through the Chinese Center for Disease Control and Prevention for researchers who meet the criteria for access to confidential data.
